# Gender Difference in Relationship between Health-Related Quality of Life and Work Status

**DOI:** 10.1371/journal.pone.0143579

**Published:** 2015-12-02

**Authors:** Jin-Won Noh, Jinseok Kim, Jumin Park, Hyun-jung Kim, Young Dae Kwon

**Affiliations:** 1 Department of Healthcare Management and Institute of Global Healthcare Research, Eulji University, Seongnam-Si, Gyeonggi-Do, Republic of Korea; 2 Department of Social Welfare, Seoul Women's University, Nowon-Gu, Seoul, Republic of Korea; 3 University of Maryland School of Nursing, Baltimore, Maryland, United States of America; 4 School of Business, Ewha Womans University, Seodaemun-Gu, Seoul, Republic of Korea; 5 Department of Humanities and Social Medicine, College of Medicine and Catholic Institute for Healthcare Management, the Catholic University of Korea, Seocho-Gu, Seoul, Republic of Korea; Nathan Kline Institute and New York University School of Medicine, UNITED STATES

## Abstract

This study investigated the association of employment status with health-related quality of life in adult Koreans, as well as the gender difference in the relationship, using a large, nationally representative sample. Using data from the Korea Health Panel survey, we examined the relationship between quality of life measured by EQ-5D and work status among Korean adults. We also tested whether and how the relationship between quality of life and work status differed by gender. Quality of life among working adults was better than among non-working adults. The gap between the two groups was larger among male than female participants. Further, the gender differential effect was larger in the 41–60-year-old age group than in the 18–40-year-old and 61-or-older groups. Being employed has a positive relation to quality of life among adults. Work status plays a more important role in quality of life for men than for women, especially for the working elderly men than working elderly women.

## Introduction

Employment is an essential element of an adult’s life, providing not only income but also a sense of engagement, role identification, and physical and mental stimulation [1,2]. Therefore, unemployment has been attributed as a possible source of adverse consequences for health-related quality of life (HRQOL), which reflects the physical, psychological, and emotional dimensions of well-being [2]. A growing body of evidence consistently demonstrates that unemployment has a substantial, negative effect on quality of life in the general [3–6].

Gender differences in the correlates of employment status have been interpreted as the cumulative reflection of gender-asymmetric roles across the life course. In the 1940s, women began to join men in the labor force, in a time of traditional gender norms with regard to male breadwinning and female homemaking. Upon marriage or the birth of their first child, women who could afford to typically left the labor force, and they often returned to work when the children were school age or older [7]. Men experienced a more continuous employment career in the breadwinning role, in which employment was recognized as their central role in their families’ lives [8]. However, in the second half of the twentieth century, women’s participation in the labor market increased. In 2014, the overall global female labor force participation rate stood at 50.3 percent, meaning that half of all women of working age were either employed or looking for work [9]. In the United States, 57.7 percent of women were in the labor force in 2012. Meanwhile, men’s proportional participation in the labor force, which had always been much higher than women’s, edged down from 70.5 percent to 70.2 percent. Women have, in addition, increasingly attained higher levels of education, and women’s earnings relative to men’s have also grown over time [10]. Given the contingent nature of women’s participation in the labor force as well as the phenomenal changes in gender expectations, however, most of what we know about employment status is based on studies of men.

Several studies have explored the effects of gender on work and quality of life; however, the results were controversial [3,11,12]. Being unemployed generally affected men more severely than women [12]. When confronted with unemployment, men are more likely than women to express greater use of self-blame, other-blame, and catastrophizing, and to report lower use of perspective-taking strategies [3]. In contrast, Brereton et al. reported unemployed males and females to be equally dissatisfied, but part-time employment had a significantly, negative impact on life satisfaction only for males [11].

In addition, age has been hypothesized as a potential moderator of the relationship between work status and quality of life. In particular, for most adults older than age 60, retirement has been considered an important life transition. The subjective developmental and social psychological transformation might be related to quality of life [13]. As life span increases, many older adults keep continue to have jobs after the age of retirement. [14]. Therefore, quality of life related to employment status might different below and above the conventional retirement age. The age-related difference between the employment status and quality of life, however, remains unclear.

With respect to the relationship between HRQOL and employment status in adults, very few studies have been conducted. In the present study, we conducted a refined analysis including the influence of gender on the association between employment status and HRQOL, whereas previous investigations have focused mostly on the quality of life effects of employment status, without regard for gender differences. The majority of studies have focused on Western developed countries, and in-depth investigations among Asian populations have been limited. Furthermore, little research has used national data. Therefore, the purpose of this study was to examine the association between employment status and HRQOL, and to test if there is difference regarding gender in adults, using a large, nationally representative sample in South Korea.

## Methods

### Data and Subjects

We used data from the Korea Health Panel (KHP) survey. The KHP is conducted by the Korea Institute for Health and Social Affairs and the National Health Insurance Service to provide information on dynamic changes in health-care service utilization patterns and expenditures. Data were collected every year from the same group of people beginning in 2008. Euro QOL five dimensions questionnaire (EQ-5D) was measured only in 2009, 2010, and 2011 while other variables such as work status and other covariates were measured every year from 2008. We did not include data from 2008 because the EQ-5D was not included in that year. Thus, the current analysis utilized adult (age 18 or older) data from the 2009 (N = 14,570), 2010 (N = 13,526), and 2011 (N = 12,803) KHP surveys. KHP data were made available to the public via their website (https://www.khp.re.kr:444/ver_2/03_data/data01.jsp). Our study procedures were reviewed and approved by the Institutional Review Board of Seoul Women’s University (IRB-2014A-20). We received a waiver of informed consent because the data were obtained from a public database.

### Variables

Employment status was coded as 1 if respondents answered that they were working and 0 otherwise. We did not distinguish retirees from non-retirees in this analysis so that those who were coded as unemployed might include retirees as well. HRQOL was measured using EQ-5D, which was relatively short and widely used in health related literature. Participants were asked to rate their level of mobility, self-care, usual activity, pain/disability, and anxiety/depression in the EQ-5D questionnaire using a 3-point Likert scale. There are about 13 countries including UK, USA, and Japan that have well-established weight system to calculate the EQ-5D scores from the scale. In calculating the HRQOL scores using EQ-5D, we utilized the South Korean population-based preference weights for EQ-5D suggested by Lee et al [15] in which a higher score of EQ-5D indicated better HRQOL of a respondent. We used relevant covariates including age, marital status, education level, alcohol drinking, smoking, and the presence of chronic disease; age groups (young age: 18–40, middle age: 41–60, old age: over 61 years old), marital status (having spouse or no spouse), education level (less than elementary school, middle/high school graduation or more than graduating from university), alcohol drinking (yes or no), smoking (yes or no), the prevalence of doctor-diagnosed chronic disease (yes or no).

### Statistical Analysis

We conducted a series of descriptive analyses of the data to examine the sample characteristics. Considering the repeatedly measured nature of the panel data, a set of random effect multilevel models of panel data were estimated to test the relationship between quality of life and work status after controlling for socio-demographic and health related [16]. The moderating effect of gender on the relationship between quality of life and work status was tested by examining the significance of the cross-product term between gender and work status in the same model. Stata version 13.1 (Stata Corp LP, College Station, Texas) was used to estimate the analysis models.

## Results

The socio-demographic characteristics of the study participants are presented in Table 1. There were 14,570 adults aged 18 or older included in the study of year 2009, of which 7,587 (52.1%) were male and 8,636 (59.3%) were employed, and the mean age was 47.8 years (SD = 16.6). Table 1 also presents the summary statistics of the EQ-5D scores by year and by age group. The EQ-5D scores did not change substantially over time, and they were on average higher for males than females throughout the three years included in this analysis (t = 11.1, p < .001 in 2009; t = 9.6, p < .001 in 2010; t = 9.1, p < .001 in 2011) (Table 1).

**Table 1:** Charactersitics of study sample stratified by gender group.

Variable	Total		Male		Female	
	(N = 14,570)		(N = 7,587)		(N = 6,983)	
	Mean	(SD)	Mean	(SD)	Mean	(SD)
EQ-5D scores						
Year 2009 (N = 12,523)	0.91	(0.08)	0.92	(0.07)	0.90	(0.09)
Year 2010 (N = 11,932)	0.91	(0.09)	0.92	(0.08)	0.90	(0.09)
Year 2011 (N = 11,427)	0.91	(0.09)	0.92	(0.08)	0.90	(0.092)
Age						
Year 2009 (N = 14,570)	47.8	(16.6)	47.1	(16.1)	48.4	(16.9)
Year 2010 (N = 13,526)	49.0	(16.6)	48.3	(16.1)	49.7	(16.9)
Year 2011 (N = 12,803)	50.1	(16.5)	49.4	(16.1)	50.8	(16.8)
	N	%	N	%	N	%
Work status (employed)						
Year 2009 (N = 14,570)	8,636	59.3	5,074	72.8	3,562	47.0
Year 2010 (N = 13,526)	7,990	59.1	4,654	72.0	3,336	47.3
Year 2011 (N = 12,803)	7,646	59.7	4,428	72.6	3,218	48.0

SD, standard deviation; EQ-5D, Euro QOL five dimensions questionnaire

**Fig 1:**
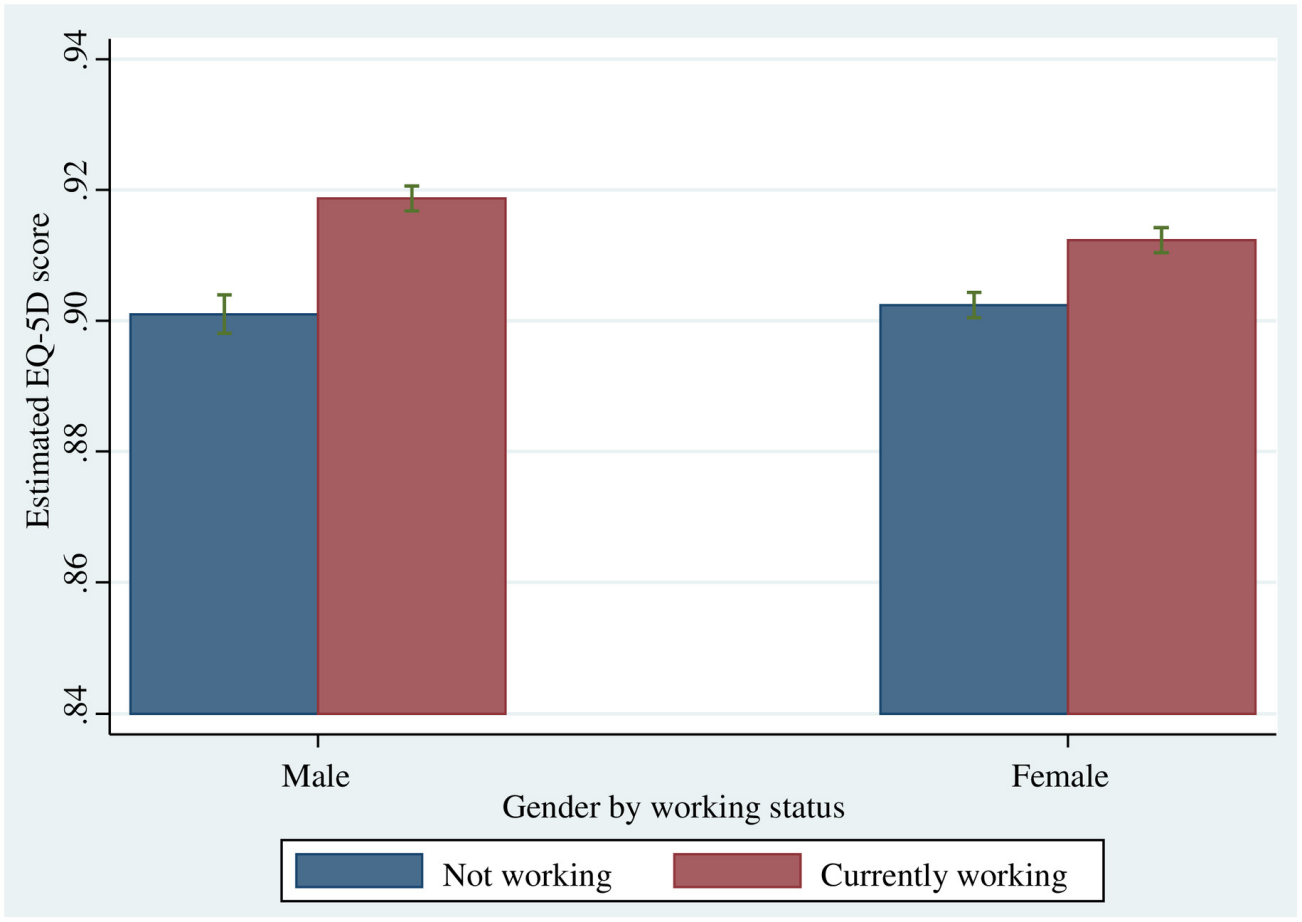
Relationship between quality of life and work status by gender.

Table 2 summarizes the random effect panel regression model of quality of life measured by EQ-5D, with work status and gender as explanatory variables. Results from Model 1 show that, in general, those who were working presented a higher level of quality of life than those who were not working [B (SE) = 0.013 (0.001), p < .001]. Further, we tested whether the relationship between quality of life and work status was influenced by gender, and the results are shown for Model 2 in Table 2. The association between quality of life and work status was stronger for males than females [B (SE) = -0.008 (0.002), p < .001] (Table 2). Fig 1 presents the specific gender differential relationship between quality of life measured by EQ-5D and work status. The difference between the working and non-working groups in terms of quality of life measured by EQ-5D was bigger for males than females (Fig 1).

**Table 2:** Random effect panel regression model of EQ-5D.

	Model 1			Model 2		
	B*	SE (B)	95% CI	B*	SE (B)	95% CI
Work	0.013**	0.001	(0.011, 0.015)	0.018**	0.002	(0.014, 0.021)
Female				0.001	0.002	(-0.002, 0.005)
(Work) X (female)				-0.008**	0.002	(-0.012, -0.004)
Intercept	0.887**	0.002	(0.882, 0.891)	0.886**	0.003	(0.881, 0.891)
	SD	Chi^2		SD	Chi^2	
Random intercept	0.05	4109.80**		0.05	4100.90**	

B1, regression coefficient; SE, standard error; CI, confidence interval; SD, standard deviation.

*Coefficients are adjusted for age, marital status, education, alcohol drinking, smoking, and any diagnosis of chronic disease.

**p < .001

**Fig 2:**
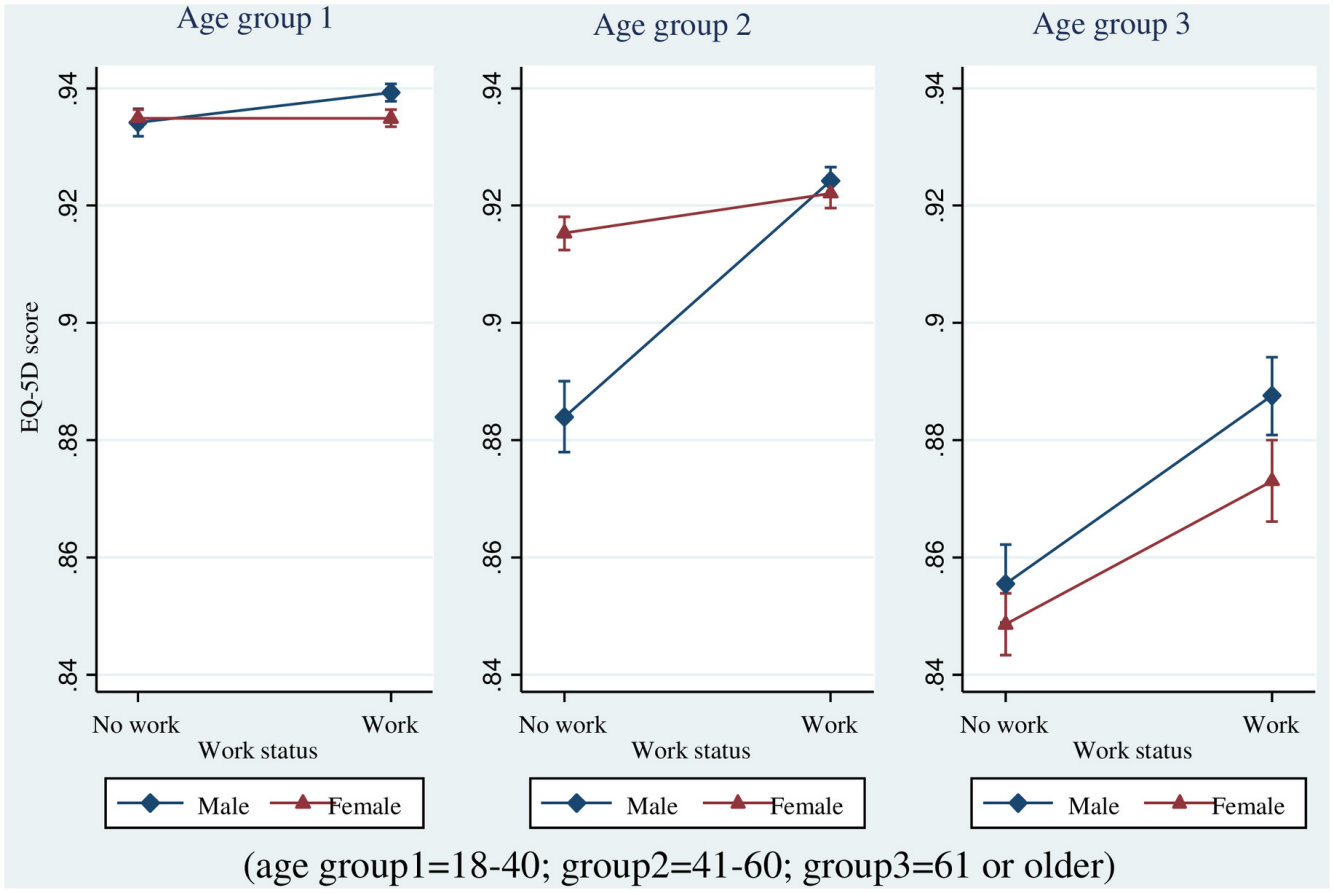
Relationship between quality of life and work status by gender by age group.

Furthermore, we examined the relationship between EQ-5D and work status, as affected by gender and by age groups of 18–40 years old, 41–60 years old, and 61 years or older. Fig 2 showed the age group differential relationship between work status and quality of life, with respect to gender (Fig 2). As displayed in Table 3, the gender difference in the relationship between work status and quality of life was strongest for participants who were 41–60 years old [B (SE) = -0.034 (0.004), p < .001], followed by those who were 18–40 years old [B (SE) = -0.005 (0.002), p = .001]. For those who were older than 61, the relationship between work status and quality of life was not different by gender [B (SE) = -0.008 (0.006), p = .161] (Table 3). We tested a three-way interaction among work status, gender, and age group, which was significant as well [chi^2^ (df = 2) = 43.29, p < .001].

**Table 3:** Random effect panel regression model of EQ-5D by age group.

Age group	18–40 years old			41–60 years old			61 or older		
	B*	SE (B)	95% CI	B*	SE (B)	95% CI	B*	SE (B)	95% CI
Work	0.005***	0.001	(0.002, 0.008)	0.040***	0.003	(0.034, 0.047)	0.032***	0.004	(0.024, 0.040)
Female	0.001	0.001	(-0.002, 0.004)	0.031***	0.003	(0.025, 0.038)	-0.007***	0.005	(-0.016, -0.002)
(Work) X (female)	-0.005**	0.002	(-0.008, -0.002)	-0.034***	0.004	(-0.041, -0.026)	-0.008	0.006	(-0.019, 0.004)
Intercept	0.915***	0.008	(0.900, 0.930)	0.864***	0.004	(0.856, 0.872)	0.874***	0.007	(0.860, 0.887)
	SD	Chi^2		SD	Chi^2		SD	Chi^2	
Random intercept	0.020	658.75***		0.039	1402.40***		0.077	1172.15***	

B, regression coefficient; SE, standard error; CI, confidence interval; SD, standard deviation.

*Coefficients are adjusted for marital status, education, alcohol drinking, smoking, and any diagnosis of chronic disease.

**p < .01

***p < .001

## Discussion

The perception of quality of life is affected by various aspects such as people’s physical and mental health, family and social relationship, or communities [17–19]. Thus, most researchers have adopted multidimensional approaches, as evidenced by the EQ-5D questionnaire employed in this study. Data from previous reports show that employment may affect both physical and psychological health [20,21] as well as leisure [22], and social participation [23]. More specifically, some studies presented consistent findings suggesting that the level of the HRQOL is significantly different by age groups, educational levels, and occupation [24]. Furthermore, it has been shown that the main influencing factors on HRQOL differ by gender, being economic activity the stronger influence for men, and educational level, psychological and physical stress, and unmet basic needs, the main factors for women [25]. According to a previous study, socio-environmental determinants such as education, economic status, occupation, and family relationship played a more important role in women's health-related quality of life, compared to men's. This study has emphasized a fragmented approach to health vulnerable targets, with improvement in social policies and suggestions about research projects for improving women’s health life and HRQOL [26]. In contrast, our study suggests that work status has a strong association with men’s quality of life, whereas it does not significantly correlate to women’s quality of life. In addition, work status had a positive relation with the quality of life of working elderly. After determining the relationships between quality of life and three significant factors, work status, gender, and age, we additionally analyzed the gender difference according to three age groups (young adult, middle adult, old adult): 18–40 years old, 41–60 years old, and 61 years or older. For those older than 60, the gender difference in the relationship between work status and quality of life was not significant, in contrast to those who were 18–40 and 41–60 years old. To recapitulate the essential finding, there is no significant difference between men and women at the age of full retirement, but the gender difference in the working age is evidently identified. Similar results where evidenced in a study of Chinese workers in production age, in which HRQOL was found to be greater among employed than non-employed people, and among men than women [27]. This could be interpreted as resulting from a men’s perception of responsibility [8], although women’s labor force participation rate increased.

In Fig 2, it appeared that 18–40 year olds are better off in terms of quality of life; and work offered gains to males but didn’t make much difference to females. It could be that there is little expectation regarding employment from 18–40 year old females in the work force. 41–60 year old males were a much clearer gain with work, whereas wom+en gain were moderate, albeit positive. Finally, 61+ year old males continued to see gains, while 61+ year old females experienced the biggest gains with work, compared to the other two females’ age groups. These findings suggest that having a job may be more important to elderly women than to the youngers. In the case of Korea, many elderlies have retired without proper retirement preparation, such as many women who live alone or women who are responsible for the household economy at their elderly age [28]. Thus, we hypothesized that the financial situation of elderly women may be more precarious than that of elderly men, influencing women’s perception of employment after the retirement age. In particular, both the average life expectancy and periods of living in celibacy of elderly women are longer than elderly men. Therefore, our results support the development of policies to expand training opportunities regarding economic activity and social participation for elderly women.

Future research should be conducted to investigate the important moderating factors of work status and quality of life in addition to gender, such as the employment status of the spouse and the presence of a dependent child. SJ Lim revealed that family relations such as family rituals and parent-child relationship might determine the level of perceived HRQOL [24]. Unfortunately, we could not address that possibility with this study. Additionally, rather than setting work status as a dichotomous variable—“have a job” or “do not have a job”—it would be more meaningful to study quality of life according to the type of work status, such as full retirement, part-time retirement, and job mobility. Additionally, further research to determine the important influences on quality of life for retirees and middle-aged or elderly people who will retire soon could help in developing policies to improve their quality of life. As the average age of retirement in Korea is increasing, we need to design new policies for elderlies, especially those older than 50, considering work status and gender.

This study has some limitations. EQ-5D could not be measured for all the respondents due to attrition and no responses, which may cause potential bias in results. We compared missing and valid groups in terms of their socio-demographic characteristics such as work status, age, and gender, and found that respondents in the missing group were younger, more likely to be female, and less likely to work than those in the non-missing group. Furthermore, the cross-sectional study design does not allow analysis of cause and effect relationships.

## Conclusions

This study evidenced a positive relation between quality of life and being employed, for men in all ages. Although the women’s participation in the labor market is rapidly expanding, men’s employment status appears to be significantly more related to quality of life compared with women’s.
